# Molecular Docking and Antihypertensive Activity of *Eupalitin 3-O-β-D-galactopyranoside* Isolated from *Boerhavia diffusa* Linn

**DOI:** 10.3390/pharmaceutics16121628

**Published:** 2024-12-23

**Authors:** Ilyas Uoorakkottil, Rashid Koottangodan, Kamal Y. Thajudheen, Saad Ali Alsheri, Mohammed Muqtader Ahmed

**Affiliations:** 1 Department of Pharmacognosy and Phytochemistry, Moulana College of Pharmacy, Perinthalmanna 679321, KL, India; 2 Department of Pharmacology, Moulana College of Pharmacy, Perinthalmanna 679321, KL, India; rsk4ma@gmail.com; 3 Department of Pharmacognosy, College of Pharmacy, King Khalid University, Abha 61441, Saudi Arabia; kthajudeen@kku.edu.sa (K.Y.T.); salshhri@kku.edu.sa (S.A.A.); 4 Department of Pharmaceutics, College of Pharmacy, Prince Sattam Bin Abdulaziz University, Alkharj 11942, Saudi Arabia; mo.ahmed@psau.edu.sa

**Keywords:** *Boerhavia diffusa* Linn., *eupalitin 3-O-β-D-galactopyranoside*, UV spectroscopy, NMR, UPLC-MSMS, pharmacokinetic study, antihypertensive activity

## Abstract

**Background:** Angiotensin-converting enzyme (ACE) is a key regulator of blood pressure, and ACE inhibition is an essential part of the treatment of hypertension. We used a molecular docking approach to find the interaction of ACE with an active flavonoid isolated from *Boerhavia diffusa* Linn, *eupalitin 3-O-β-D-galactopyranoside*, which leads to potential antihypertensive effects in methyl predenisolone-induced hypertensive rats. Additionally, the pharmacokinetic parameters of this compound are assessed. **Methods:** *eupalitin-3-O-β-D-galactopyranoside* was isolated from leaves of *Boerhavia diffusa* by sedimentation method. The compound was characterized by UPLC-MSMS, NMR, and UV spectroscopy to confirm the identity of the compound. Hypertension was induced in rats with methyl predenisolone (5 mg/kg/day) for 14 days. Systolic and diastolic blood pressure effects of *eupalitin 3-O-β-D-galactopyranoside* were assessed using a tail-cuff method. The blood plasma data for oral administration were used to determine various pharmacokinetic parameters from the bioavailability and serum concentration. **Results:** In methyl predenisolone-induced hypertensive rats, both systolic and diastolic blood pressures were significantly lower than that of the vehicle with treatment from *eupalitin 3-O-β-D-galactopyranoside* (*p* < 0.01). **Conclusions:** The pharmacokinetic process showed the moderate bioavailability of the compound; *eupalitin 3-O-β-D-galactopyranoside* induces powerful antihypertensive activity in methyl predenisolone-induced hypertensive rats, implying potential clinical application as a new therapeutic drug for hypertension.

## 1. Introduction

**Figure 1:**
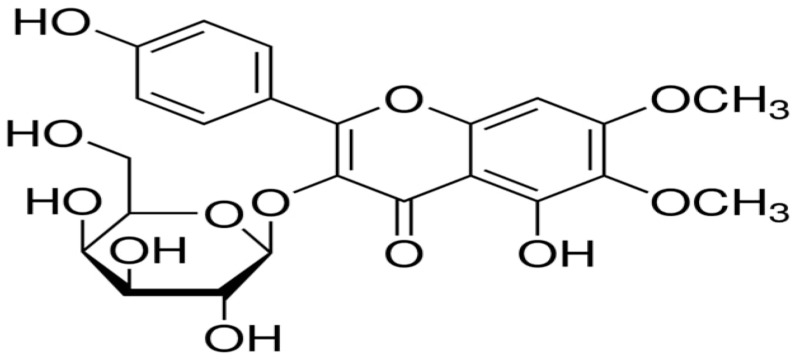
The chemical structure of eupalitin-3-*O-β-D*-galactopyranoside.

Hypertension, more commonly known as high blood pressure, is a chronic medical condition wherein the blood pressure remains raised in the arterial blood vessels. It is severe public concerns worldwide and affects approximately an estimated 1.13 billion people [1]. According to the Global Burden of Disease Study, over the last couple of decades, there has been a sharp rise in the prevalence of hypertension, which is largely driven by senior populations and changes in lifestyle variables such as food, physical sedentary behavior, and raised body mass index (BMI). Cardiovascular complications like myocardial damage or heart attacks, stroke, and renal failure are developed by persistent hypertension. Hence, it is one of the foremost causes of morbidity and mortality. The annual estimation of the WHO reported that hypertension causes 9.4 million fatalities, accounting for about 12.8% of the global mortality rate [2]. The rennin angiotensin aldosterone system, or RAAS, is crucial for modulating clinical hypertension by regulating vascular flexibility and balancing the salt content. Additionally, angiotensin II affects heart and kidney vasculature function and increases the reabsorption of salt and water, all of these subsidizing the increase of blood pressure [3]. Disruption in these pathways can result in the development of chronic hypertension and organ damage. The angiotensin-converting enzyme (ACE) is a two-domain dipeptidylcarboxypeptidase that has a direct participation in the control of blood pressure by hydrolysis of angiotensin I to produce angiotensin II [4]. Angiotensin II is a powerful vasoconstrictor and stimulant for the secretion of aldosterone, an event causing an elevation of blood pressure and fluid retention. The deregulation of this system will lead to cardiovascular disorders such as heart failure, myocardial infarction, and hypertension [5]. ACE inhibitors, such as captopril, enalapril, and lisinopril, are used to prevent the generation of angiotensin II in the treatment of these disorders [6]. Medications of this kind are quite effective; nevertheless, they have certain limitations, including potential adverse effects and individual sensitivity to medicines [7]. Therefore, it requires searching for cheaper and non-toxic natural products. Since many therapeutic molecules emerge from natural products, they have been used as a source for active modern medicines [8]. In the past few decades, a lot of investigation has been done on medicinal plants for treating cardiovascular diseases, providing insight into natural sources for ACE inhibition [9]. *Boerhavia diffusa* is an herbaceous *Nyctaginaceae* family member with a long history of use by Brazil’s indigenous and tribal peoples. In particular, the roots and leaves of this plant have been widely used in folk medicine to treat several illnesses, including those affecting the gastrointestinal tract. This plant has been reported to exhibit a wide range of bioactivities, including behavioral and neuroendocrine effects [10], antidepressant activity [11], anti-angiogenic properties [12], as well as anticonvulsant and antiepileptic effects [13]. It also demonstrates anti-inflammatory activity [14], potential benefits in prostatic hyperplasia [15], and genotoxic and antigenotoxic properties [16]. Additional reported activities include thrombolytic, cytotoxic, and antimicrobial effects [17], protection against arsenic trioxide-induced cardiotoxicity [18], antiurolithic effects [19], and antihyperglycemic and renoprotective properties [20]. Further studies have shown its efficacy in alleviating acetaminophen-induced liver toxicity [21], antiproliferative and antiestrogenic effects [22], immunomodulatory and anti-metastatic activities [23], and radio-protective properties [24]. Moreover, the plant has demonstrated potent anti-breast cancer effects [25], intestinal activity [26], immunomodulatory effects [27], anti-diabetic activity [28], Ca^2^⁺ channel antagonistic properties [29], chemopreventive action [30], and human oral cancer cells [31]. Within the plant kingdom, flavonoid glycosides represent a diverse group of naturally occurring compounds with a wide distribution and extensive pharmacological activities. These molecules have several health advantages, such as anti-inflammatory, antioxidant, and cardiovascular protective properties [32]. In flavonoid glycosides, flavonoids are conjugated with sugar moieties, which enhance their bioavailability by improving absorption and stability. *Eupalitin 3-O-β-D-galactopyranoside*, a flavonoid glycoside isolated from *Boerhavia diffusa*, has shown immunosuppressive and hepatoprotective activity [33,34]. The aim of this study was to isolate *eupalitin 3-O-β-D-galactopyranoside*, a flavonoid glycoside from *Boerhavia diffusa*, through the sedimentation method. The antihypertensive properties of the isolated compound will be determined using the hypertensive model of rats induced with methyl prednisolone. Moreover, molecular docking studies will help predict the interaction between *eupalitin 3-O-β-D-galactopyranoside* and important blood pressure-regulating molecules. Under this research protocol, the antihypertensive activity of *eupalitin 3-O-β-D-galactopyranoside* will be confirmed, and elucidation of pharmacological properties using in vivo and computational studies will be carried out so it could be used for the future therapeutic management of hypertension and cardiovascular disorders. The chemical structure of eupalitin-3-*O-β-D*-galactopyranoside is shown in Figure 1.

Molecular docking is a specifically attractive tactic in the lead identification phase of a compound. It helps researchers screen large databases in a short time, select the probable molecule, and enhances the lead molecule for better efficacy and safety [35]. In the case of hypertension, flavonoid glycosides offer promising avenues for developing novel ACE inhibitors. By elucidating the binding interactions and evaluating pharmacokinetic properties, these studies enhance our understanding of the therapeutic potential of natural compounds. An in vivo screening is of utmost importance in depicting the therapeutic potency and safety profile of handling a new compound. The initial signs of its activity can be presumed from the in vitro studies, while in vivo tests are crucial in understanding the action of the compound in a living organism. These studies assist in determining the pharmacokinetic characteristics, such as bioavailability, metabolism, and efficacy of the compound in a physiological framework. The data obtained from the molecular docking and in vivo studies provide a robust framework for the development of a new entity with prominent ACE inhibition properties. The effectiveness and safety of potential ACE inhibitors in a biological system can be validated by in vivo study, while molecular docking deals with insights into the mechanisms of action and guides further optimization of compounds. In total, the outlined strategies contribute to discovering new opportunities in the treatment of hypertension and other cardiovascular diseases.

## 2. Materials and Methods

Isolated and purified *eupalitin 3-O-β-D-galactopyranoside* came from Hamdard Laboratory (New Delhi, India). LC-MS grade acetonitrile, formate, and methanol came from Sigma (IOL Chemical Ltd., Mumbai, India). A Milipore system was used to purify water (Bedfrod, MA, USA). Methanol HPLC grade (SD Fine Chemicals, Mumbai, India) was used as a solvent for the preparation of standards and samples. Toluene, acetone, and water (CDH Labs, Mumbai, India) were used as mobile phases for HPTLC analysis. 2-Amino ethyl diphenylborinate was procured from Sigma-Aldrich Co. LLC (St. Louis, MO, USA). All solutions used for the analysis were filtered through a 0.22 μm syringe-driven filter (HIMEDIA, Mumbai, India)

### 2.1. Isolation and Characterization of Eupalitin 3-O-β-D-galactopyranoside

Dried powdered plant material was extracted three times by using 80% methanol at 40 °C. The combined hydro-alcoholic extracts were pooled, filtered, and concentrated in a rotary evaporator under reduced pressure to get residues (28%). The crude extracts obtained were portioned-fractionated with hexane (3 × 250 mL), chloroform (3 × 250 mL), and ethyl acetate (3 × 250 mL). The dried ethyl acetate residue (15 g) was thoroughly mixed in hot methanol, stored overnight in the refrigerator, and filtered. The solid was separated and crystallized multiple times with hot methanol to yield *eupalitin 3-O-β-D-galactopyranoside* (0.1578% *w*/*w*). The ethyl acetate residue was re-dissolved in HPLC-grade methanol. A standard solution of *eupalitin 3-O-β-D-galactopyranoside* was prepared in methanol. Both the standard and ethyl acetate residue were applied onto pre-activated HPTLC plates using a micro-liter syringe. The plates were developed in a solvent mixture (toluene: acetone: water, 4:15:1) and dried. Visualization was done under UV light (366 nm) and by spraying with 2-Amino ethyl diphenyl borinate reagent for flavonoid detection. The R_f_ values (0.53) of the spots from the unknown sample were compared with the standard to confirm the presence of *eupalitin 3-O-β-D-galactopyranoside***.** The ethyl acetate residue of *eupalitin 3-O-β-D-galactopyranoside* was dissolved in deuterated chloroform [CDCl_3_] and the concentration of sample (10 mg/mL in sample). NMR spectra were recorded on a Bruker AVANCE III 500 MHz spectrometer. Proton NMR (1H) was done at 90° pulse, and a relaxation delay of 2 s after 32 scans was performed to facilitate signal assignments. HPTLC and *UPLC-MS* were recorded to confirm the structure. UPLC-MSMS was performed with a binary solvent delivery pump, an autosampler, and a PDA detector of an Acquity UPLC system manufactured by Waters Corporation (Milford, MA, USA); data were acquired and processed using Empower software 2.0. The UPLC system utilized a C18 column (150 mm × 2.1 mm, 3.5 µm). The mobile phase consists of water with 0.1% formic acid (A) and acetonitrile with 0.1% formic acid (B). The gradient read as following values: 0–5 min, 5% B; 5–15 min, 5–95% B; 15–20 min, 95% B; 20–21 min, 95–5% B; 21–30 min, 5% B. With an injection volume of 10 µL, the flow rate was 0.3 mL/min. The mass spectroscopic analysis was carried out using a Q-TOF with an ESI source in positive mode. The source temperature was 101 °C, while desolvation temperature was 351 °C. Capillary and cone voltages were 2.95 kV and 40 V, respectively. The full scan (*m*/*z* 100–1000) and MRM modes were used for data acquisition.

### 2.2. Molecular Docking Studies

The structure optimization of *eupalitin 3-O-β-D-galactopyranoside*, captopril, and PDB ID: 1J37 (ACE) was performed using Schrödinger software [LLC, New York, NY, USA, 2015]. The OPLS3e force field was used to prepare the ligands using LigPrep tool to generate three-dimensional conformers, ionization states, and partial charges. Ligand geometry was done using Prime at a preset convergence threshold of 0.05 kcal/mol/Å. The protein (PDB ID: 1J37) was prepared with the Protein Preparation Wizard, which included the addition of H atoms, bond order assignments, and a minimization step to eliminate sterically hindered regions, although some flexibility was allowed in the active site. For docking, a grid box was prepared to enclose the active site of the ACE protein. The grid was set at 30 × 30 × 30 Å^3^ with 0.375 Å grid spacing to ensure the binding pocket was sufficiently covered. The Site Map tool was utilized to establish the binding site for the best docking position. The docking procedure and algorithm was achieved by using Glide in standard precision (SP) mode. Ligands were assigned flexibility in the rotatable bonds, while the protein was held rigid throughout the process. Twenty poses per ligand were created, and the one with the highest value of Glide score was selected. Glide score gives a binding log by evaluating the electrostatic interactions, van der Waals interactions, and hydrogen bonding interactions. Captopril and *eupalitin 3-O-β-D-galactopyranoside*, an identified ACE inhibitor, have been redocked into PDB ID: 1J37 to verify the docking process. An RMSD of less than 2 Å was obtained when comparing the re-docked pose to the crystallographic stand.

### 2.3. Measurement of ACE Inhibitory Activity

To assess the ACE inhibitory potential of *eupalitin 3-O-β-D-galactopyranoside* and captopril, an ACE inhibition assay was performed following their standard methods. The assay included the preparation of stock solutions of both captopril (standard ACE inhibitor) and *eupalitin 3-O-β-D-galactopyranoside* in high concentrations (1000 µM), followed by dilution to give various concentrations (25, 50, and 100 µg/mL). The enzyme solution and substrate were prepared under standard protocols, and the assay was performed in 96-well plates wherein the ACE enzyme and inhibitor were mixed with the substrate. The reaction was incubated at 37 °C for 30 min, after which the ACE activity was determined by measuring the absorbance at 340 nm that correlated with the product formation and enzyme activity [36]. The IC₅₀ values that specify inhibition at 50% concentration were generated from concentration-inhibition data. The percentage inhibition was calculated using the formula:

Percentage Inhibition (%) = (A − C)/ (A − B) ∗ 100

where A is the absorbance of the negative control (substrate and enzyme), B is the absorbance of the blank (buffer only) and C is the absorbance of the sample (enzyme, substrate and sample). For the positive control, captopril was used instead of a sample and was considered as C.

### 2.4. Method of Validation and Pharmacokinetic Studies of Eupalitin by UV—Visible Spectroscopy

In accordance with the ICH guidelines Q2 (R1) (2005) [37] standard calibration curves, regression equations, and values for LOD and LOQ for each compound were validated with UV spectrophotometric method. Various parameters, such as linearity, precision, limit of detection, and limit of quantification, were considered for the method validation. *Eupalitin 3-O-β-D-galactopyranoside* was dissolved in 5% CMC-Na and prepared in 10, 20, and 40 mg/kg, body weight (suspension, 20 mg/mL). The rats were orally administered (10, 20, and 40 mg/Kg body weight, *n* = 3) using a 20 G gavage needle. The animals were given a standard diet 4 h after the dosing, and blood samples were collected (approximately 400 μL) from each animal and placed into heparinized tubes before the dosing and at 0, 30, 60, 120, 180, 240, 300, 360, and 420 min after the dosing. Plasma (100 μL) was separated by centrifuging blood samples at 3600 rpm for 10 min at room temperature, followed by immediate processing for the analyses [37].

### 2.5. In Vivo Antihypertensive Activity of Isolated Eupalitin 3-O-β-D-galactopyranoside

#### 2.5.1. Identification, Procurement, and Housing of Animals

Albino rats (body weight 100–200 g) were procured from the Central Animal House facility at Jamia Hamdard, New Delhi, India (Registration Number: 173/CPCSEA, 28 January 2000/Animal Ethical Approval Code: 837) and kept under standard laboratory conditions in 12 h light/dark cycle at 25 °C ± 2 °C. Pellet feed (Lipton, Calcutta, India) and unlimited water ad libitum were given to the animals. They were labeled to make it easier to identify them.

#### 2.5.2. Animal Training and Conditioning

The animals were restrained in a restrainer for 10 min every day for one week to conduct the BP measurement tests. This exercise was performed to reduce blood pressure fluctuation caused by the animal’s behavior while in the restrainer for activity measurement.

#### 2.5.3. Hypertension Induction in Normotensive Rats and Study Design

After recording the initial BP of rats, the animals were divided into seven groups of four animals each. One group was chosen as the control group. The remaining groups were given a subcutaneous injection of methyl-predenisolone acetate (20 mg/kg/week) for one week, as described by Ekerbicer, N et al. [38]. The animal study was designed to evaluate the effect of *eupalitin 3-O-β-D-galactopyranoside* and captopril in the animal model of methylprednisolone-induced hypertension. In total, 28 animals were evenly divided into 7 groups of 4 animals each to attain maximum balance of subjects across experimental conditions. Animals in group I (normal control) received normal saline by oral gavage in a dose of 2 mL/kg/week, and this was taken as the most inferior baseline for comparison. Animals in group II (hypertensive control) were injected methylprednisolone acetate (20 mg/kg/week) intraperitoneally for hypertension induction, creating a baseline model of plateau hypertension. Animals in Group III were administered with 20 mg/kg/week intraperitoneal injection of methylprednisolone treated with captopril at an oral dosage of 5 mg/kg/day. For Group IV, the basal dosage of methylprednisolone was provided along with a higher dosage of captopril (10 mg/kg/day by oral route). Methylprednisolone and gradually increasing doses of *eupalitin 3-O-β-D-galactopyranoside* (10, 20, and 40 mg/kg/day orally) were administered to Groups V, VI, and VII, allowing assessment of a dose-response to the compound, as shown in Table 1. This experimental design has enabled the evaluation of both a standard antihypertensive agent (captopril) and the test compound (*eupalitin 3-O-β-D-galactopyranoside*) as alleviating factors against methylprednisolone-induced hypertension. The inclusion of multiple treatment groups with varying doses provided insights into the relative efficacy and dose-response relationship of the interventions. Randomization was done by block randomization, ensuring without bias these allocations of animals into groups. Before intervention, baseline characteristics such as weight, age, or sex of animals were determined, and *t*-test or ANOVA was used to affirm that no significant difference between groups existed. The SYRCLE’s Risk of Bias tool analyzed potential biases across various domains like selection, performance, and detection bias; each domain was rated according to low, high, or unclear risk [39].

#### 2.5.4. Measurement of Rat’s Mean Arterial Blood Pressure

The CODA Non-Invasive Blood Pressure Recorder (Kent Scientific Corporation, Torrington, CT, USA) was used to measure mean arterial blood pressure in conscious rats using the tail-cuff method. With the rat’s tail protruding, the restrainer was inserted in the BP instrument. The rat’s restrainer was placed in the BP instrument with its tail protruding. The tail was gently pressed against a transducer membrane that was linked to the digital BP display panel. After that, the device was turned on and stabilized until a consistent pulse rate was seen. The BP recording button was pressed, and the mean arterial BP was recorded after the “pulse level ready” indicator showed. In this investigation, albino rats (body weight 150–200 g) were employed. Rats were divided into four groups, each with four individuals. After suspension in 0.1 carboxy methyl cellulose (CMC) solution, each drug (10, 20, 40, and 60 mg/kg body weight) was injected intraperitoneally. After 1 h, the mean arterial blood pressure was measured.

### 2.6. Statistical Analysis of Data

Statistical analysis was carried out by one-way analysis of variance followed by Dunnet’s multiple comparison test using GraphPad InStat 3 (Graph Pad Software Inc., San Diego, CA, USA). All values were expressed as mean ± standard error of the mean (SEM). All groups were compared with hypertensive control animals. *p*-value of <0.01 was statistically significant.

## 3. Results

### 3.1. Isolation and Characterization of Eupalitin 3-O-β-D-galactopyranoside

*Eupalitin 3-O-β-D-galactopyranoside* was isolated by sedimentation from the ethyl acetate sediment fraction of the 80% methanolic extract. HPTLC has been applied to differentiate the *eupalitin 3-O-β-D-galactopyranoside*, which was added as a reference for the analysis, where it was utilized to compare the two chromatographic spectra to identify the standard. The R_f_ values obtained from the unknown sample clearly revealed high affinity toward *eupalitin 3-O-β-D-galactopyranoside* samples to affirm the presence of the unknown sample. Additionally, the appearance of these spots concerning fluorescence under UV light and the chemical test were key elements in confirming the identity of the compound. In the UPLC-MS study, the FAB-MS exhibited (M + H)^+^ at *m*/*z* 493 as pseudo molecular ion peak. HRFAB-MS showed a molecular ion peak [M + H]^+^ at *m*/*z* 493.1331 (C19H24O12 + H = 493.1346), allowing the determination of its molecular formula as C23H24O12 and mentioned in Figure 1. At δ 8.14 d (2H, J = 8.2 Hz, H-2′, H-6′), 6.85 d (2H, J = 8.2 Hz, H-3′, H-5′), 6.89 s (H-8), 10.19 s (H-OH/H-4′), 12.57 s (H-OH/H-5), 3.90 s (H3-OCH_3_/H-6), and 3.72 s (H3-OCH_3_/H-6), the 1H NMR spectra showed aromatic proton peaks. At δ 5.41 d (1H, J = 7.6 Hz, H-1″) and 3.54–4.82 m (6H, H-2″, 3″, 4″, 5″, 6″ b, 6″ b), 1H NMR also showed signals for the sugar moiety. The previously reported *eupalitin 3-O-β-D-galactopyranoside* was a perfect match for all the physical and spectral data [40].

### 3.2. Molecular Docking Calculation

**Figure 2:**
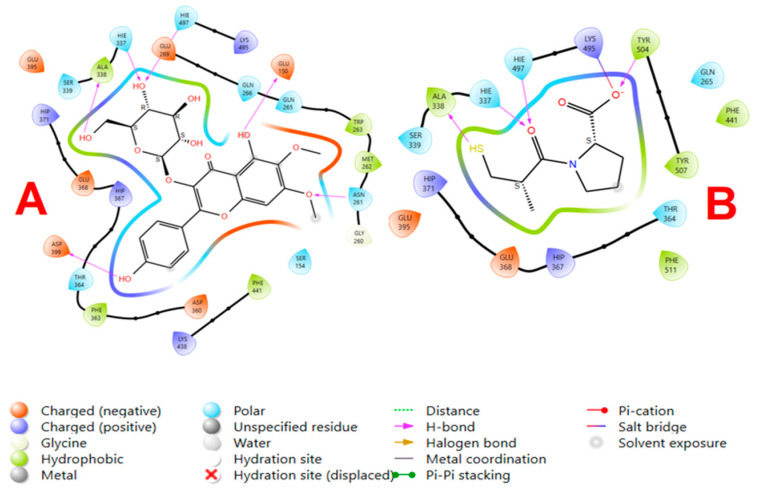
2D ligand interaction diagram of the *eupalitin 3-O-β-D-galactopyranoside* (**A**) and **captopril** (**B**) with **ACE** (PDB ID: **1J37**). The diagram illustrates the binding interactions between each ligand and ACE, showing key residues involved in the ligand–receptor binding sites.

The 2D ligand interaction diagrams of *eupalitin 3-O-β-D-galactopyranoside* and captopril were docked to a target protein (PDB ID: 1J37), as shown in Figure 2, possession of different interaction profiles that are in accordance with their corresponding docking scores. *Eupalitin 3-O-β-D-galactopyranoside* with docking score −7.98 kcal/mol wraps multiple hydrogen bonds around amino acid residues, including aspartate (asp) and serine (ser), binding the compound to the active site. Also, electrostatic interactions with residues, e.g., phenylalanine (phe), enhance the binding ability of the ligand. On the other hand, the slightly higher docking score of −9.44 kcal/mol captopril also makes hydrogen bonds with similar residues but displays a stronger overall binding with the active site. The more hydrophobic interactions and an ionic interaction between the ligand’s thiol residue and a positively charged residue contribute to the slightly higher binding strength than *eupalitin 3-O-β-D-galactopyranoside*. There are multiple reasons for the difference between the docking studies and the in vivo results from animals. *Eupalitin 3-O-β-D-galactopyranoside* might exhibit better pharmacokinetic profiles, possibly better bioavailability, stability, or absorption, and thereby lead to increased activity in vivo, even though showing reduced level of binding affinity toward ACE protein when compared to captopril standard. The compound could also exert its activity through other pathways beyond direct ACE inhibition, which could explain its better activity in animals. Thus, in vivo activity depends upon a wider range of factors than merely binding affinity, which includes the overall biological behavior of the compound. Ligand poses describe the predicted orientations and conformations of the ligands in the achieved Glide XP docking calculations (kcal/mol) in the active site of PDB ID: 1J37, as shown in Table 2. Each number (2 to 5) denotes certain spatial conformations of the *eupalitin 3-O-Β-D-galactopyranoside* in the target protein’s active site. The docking scores of these poses, arranged according to their docking scores, which measure the binding capacity and stability of the ligand inside the binding site, are considerably like that of the standard captopril docking score.

### 3.3. Measurement of ACE Inhibitory Activity

**Figure 3:**
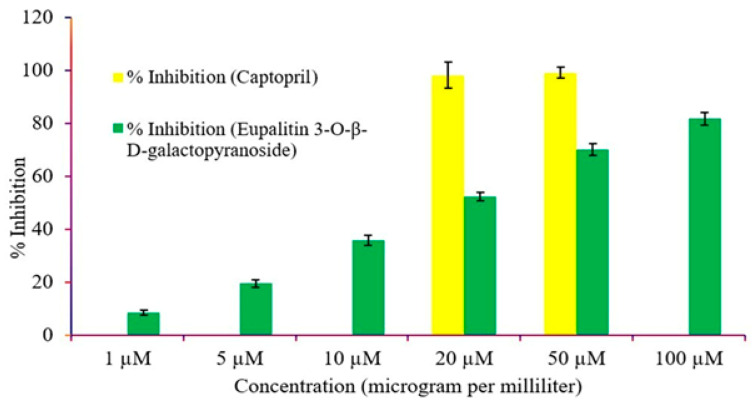
ACE inhibition activity of captopril and *eupalitin 3-O-β-D-galactopyranoside* at various concentrations.

*Eupalitin 3-O-β-D-galactopyranoside* showed a dose-dependent ACE inhibitory activity in the ACE inhibition assay, which is significant for its antihypertensive activity. Figure 3 shows low concentrations were minimally inhibiting, while high concentrations, for instance, 100 µM, produced maximum inhibition of around 82%. The IC_50_ value is estimated to be around 20 µM, which suggests that it offers moderate ACE inhibitory potential. Most potent ACE inhibition was found with this standard drug from 20 to 50 micrograms per milliliter since the increasing concentration led to a significant increase in inhibition. This has been reported by considerable literature as a potentiality of captopril and flavonoids as ACE inhibitors in the treatment of hypertension [41,42].

### 3.4. Antihypertensive Activity of Eupalitin 3-O-β-D-galactopyranoside

**Figure 4:**
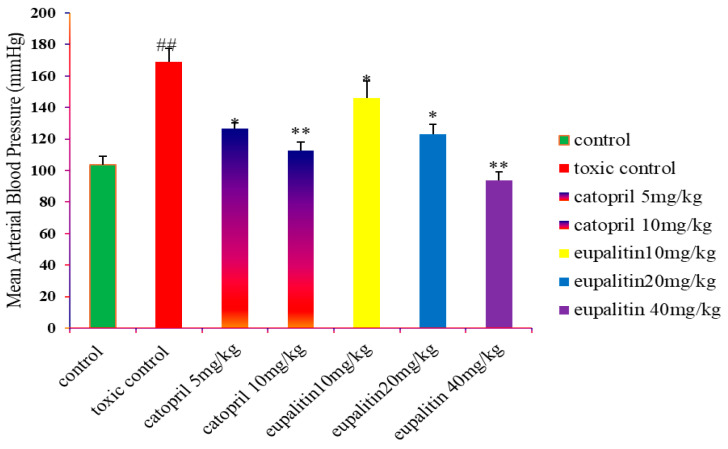
The graph shows the effect of several doses of *eupalitin 3-O-β-D-galactopyranoside* and captopril on arterial blood pressure of the rats. It indicates the changes in blood pressure caused by these compounds, *eupalitin 3-O-β-D-galactopyranoside* of doses 10, 20, and 40 mg/kg and captopril of dosages 5 and 10 mg/kg. Measurement of blood pressure had been performed using a non-invasive tail-cuff method. It was revealed that there is a drastic decrease in blood pressure with increasing doses of eupalitin 3-O-β-D-galactopyranoside, with dose levels of 20 mg/kg and 40 mg/kg, with eupalitin 3-O-β-D-galactopyranoside showing better effectiveness than captopril at the higher dose (10 mg/kg). * denotes significance, ** denotes higher significance, and ## denotes toxic significance.

The antihypertensive activity of *eupalitin 3-O-β-D-galactopyranoside* was assessed using the non-invasive tail-cuff method in experimental rats, with the aim of determining its potential for hypertension. The rats were treated with *eupalitin 3-O-β-D-galactopyranoside in* three different doses, which include 10, 20, and 40 mg/kg, besides the standard antihypertensive drug captopril at 5 mg/kg and 10 mg/kg used in the comparison. Results showed a dose-dependent decrease in blood pressure in treated rats that received the *eupalitin 3-O-β-D-galactopyranoside*, indicating antihypertensive activity of the compound. From the result, this compound at lower dose level, e.g., 10 mg/kg, which is found to exert mild action on blood pressure reduction, is equivalent to the effects of the standard drug captopril at a dose of 5 mg/kg. However, an increase in doses of *eupalitin 3-O-β-D-galactopyranoside* (20 mg/kg and 40 mg/kg) increased the antihypertensive effects, with higher doses of 40 mg/kg giving a clearer antihypertensive action than captopril at 10 mg/kg. The improved activity of *eupalitin 3-O-β-D-galactopyranoside* at high concentrations can indicate that this compound is potentially very effective for reducing blood pressure compared to potential activity with captopril at higher concentrations. Therefore, these findings indicate that *eupalitin 3-O-β-D-galactopyranoside* would serve as a promising candidate for exploration in future studies as a natural antihypertensive agent. Although this mechanism of action needs to be fully elucidated, results would suggest that *eupalitin 3-O-β-D-galactopyranoside* may ultimately act through a pathway like that which is mediated by captopril, possibly inhibiting the ACE or other cardiovascular targets. Results are shown in Table 3 and Figure 4.

### 3.5. Validation and Pharmacokinetic Parameters of Eupalitin 3-O-β-D-galactopyranoside

**Figure 5:**
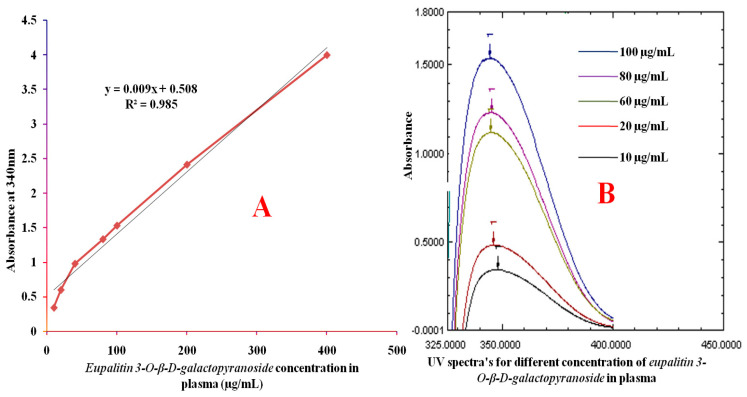
The calibration curve of the compound in plasma over the concentration range of 10–400 µg/mL the curve shows linearity with a correlation coefficient (R^2^ > 0.985) (**A**) and UV spectra of the compound at different concentrations (10–400 µg/mL) in plasma, recorded at the maximum absorption (Abs.) wavelength (**B**).

Plasma concentration by the compound was measured by UV spectrophotometry at 340 nm and validated per ICH requirements. The calibration curve demonstrated excellent linearity between concentrations 10–400 mcg/mL, with regression equation Y = 0.0009x + 0.508 and R^2^ = 0.985, as shown in Figure 5. The LOD and LOQ were found to be 3 and 5 micrograms per milliliter, respectively. The precision studies gave % RSD less than 2 for intra-day and inter-day variations, and recovery studies were between 97–99%, indicating the reliability of method.

**Figure 6:**
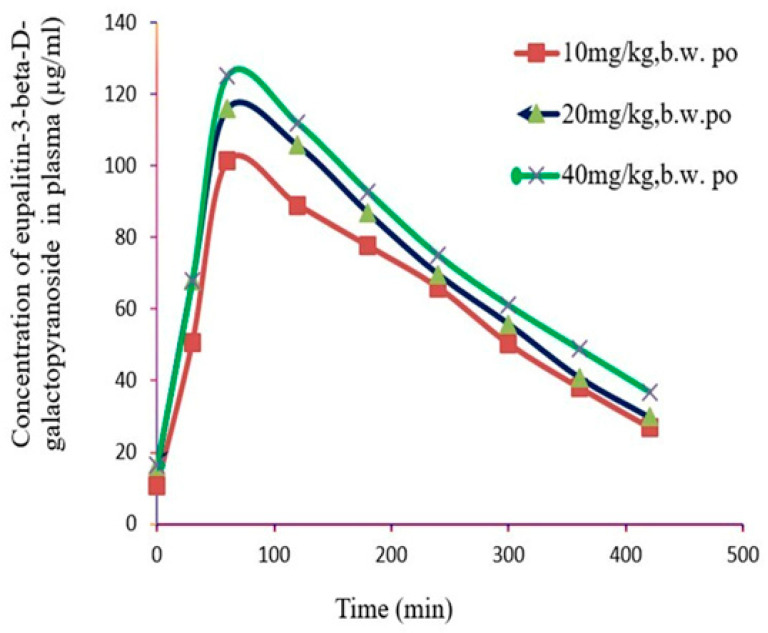
Plasma drug concentration-time profile after oral administration of *eupalitin 3-O-β-D-galactopyranoside*. The data were subjected to non-compartmental analysis based on statistical moment theory to evaluate the pharmacokinetic parameters. The figure presents the concentration of the drug in plasma over time following oral administration, showing the absorption, distribution, and elimination phases for each compound. The analysis provides insights into the pharmacokinetic behavior and compares the drug exposure profiles of *eupalitin 3-O-β-D-galactopyranoside.* b.w.: body weight; p.o.: oral administration.

The plasma concentration–time graph obtained after oral administrations were subjected to non-compartmental analyses based on the statistical moment theory, as shown in Figure 6 and Table 4. Pharmacokinetic parameters were calculated using WinNorlin 5.2 (Certara; available at www.certara.com, accessed on 20 November 2024). The parameters obtained include maximum plasma concentration (Cmax), the time to reach Cmax (Tmax), the elimination half-life (t1/2), the area under the plasma concentration–time curve from time zero to infinity (AUC_0–∞_), the area under the plasma concentration–time curve from time zero to 420 min AUC_0–7_, the mean residence time (MRT), and clearance (CL) [43].

## 4. Discussion

### Interpretation of Antihypertensive Activity

The antihypertensive activity of *eupalitin 3-O-β-D-galactopyranoside* in hypertensive animal models indicates that this compound possesses significant potential as a therapeutic agent for hypertension. The reduction in blood pressure suggests that eupalitin 3-O-β-D-galactopyranoside may influence the renin-angiotensin-aldosterone system (RAAS), a key regulator of blood pressure. *Eupalitin-3-O-β-D-galactopyranoside* was a flavonoid glycoside obtained from *Boerhavia diffusa*. Flavonoid glycosides were reported to act as an ACE inhibitor, which is widely understood for its potential antihypertensive properties, based on earlier studies [44,45] and the established pharmacological properties of flavonoid glycosides. *Eupalitin 3-O-β-D*-galactopyranoside may act by mechanisms other than ACE inhibition, including vasodilation, an anti-inflammatory mechanism, or modulation of other blood pressure-regulating molecules. To verify the conceivable effects of *eupalitin 3-O-β-D-galactopyranoside*, molecular docking studies, and in vitro ACE inhbition assay demonstrate its interaction with important molecules in the regulation of blood pressure. Captopril is a potent antihypertensive but is associated with side effects like cough and angioedema. In contrast, *eupalitin 3-O-β-D-galactopyranoside* shows lower binding affinity but may offer a safer profile with fewer side effects, making it a promising alternative for patients intolerant to captopril. Haynes et al. (2023) reported that flavonoids, a natural phenolic compound, possess several therapeutic benefits and can possibly serve as antihypertensives [46]. Flavones are a kind of flavonoid that has anti-inflammatory properties that may allow them to act as therapeutic agents for hypertension. Another study by Ahmed et al. (2022) identified diosmetin as a flavonoid with potential antihypertensive properties because of its Ca^2^⁺ channel blocking and K⁺ channel activation properties [47]. The UV, MASS and NMR spectroscopic analysis of *eupalitin 3-O-β-D-galactopyranoside* by Thajudeen et al. (2022) revealed characteristic absorption peaks associated with flavonoid contents [48]. The spectral data provided insights into the compound’s electronic transitions and molar absorptivity, confirming its presence and purity in the extraction process and supporting its potential therapeutic applications [49]. The pharmacokinetic parameters of various concentrations of *eupalitin 3-O-β-D-galactopyranoside* in rats after oral administration were presented in Table 3. The pharmacokinetics of *eupalitin 3-O-β-D-galactopyranoside* in rats was investigated in the study using UV spectroscopy. Data reveals that *eupalitin 3-O-β-D-galactopyranoside* provides valuable insights into their ADME. The Cmax (maximum plasma concentration) and Tmax (time to reach Cmax) revealed the rate and extent of absorption, which is crucial for knowing the onset of action. The elimination t_1/2_ informs the duration of action of *eupalitin 3-O-β-D-galactopyranoside*. Initial absorption phase and overall exposures of *eupalitin 3-O-β-D-galactopyranoside* were determined from the area under the curve (AUC0–420 and AUC0–∞). Mean residence time (MRT) and clearance (CL) reflect the average duration and elimination details of *eupalitin 3-O-β-D-galactopyranoside*. The collective parameters help in dose-optimizing dosage regimen, efficacy, and safety. The pharmacokinetics of *eupalitin 3-O-β-D-galactopyranoside* in rats were investigated in the study using UV spectroscopy, which generated reliable values for Cmax, Tmax, and AUC. Compared to HPLC-MS/MS that has been used by Wang et al. (2018), this technique was suited to measure the concentrations of *eupalitin 3-O-β-D-galactopyranoside* in the plasma and, therefore, allowed an extremely simple evaluation of the absorption profile. UV spectrophotometry instead of HPLC-MS/MS is advantageous by being cheaper, faster, and easier to set up and operate. It is also non-destructive, requires less technical expertise, and is available in most laboratories [50]. The molecular docking studies revealed that *eupalitin 3-O-β-D-galactopyranoside* binds effectively to the ACE protein, with binding affinities comparable to those of captopril, a well-established ACE inhibitor. PDB ID: 1J37 (ACE) was chosen as the biological target model because it gives a deep dive and well-behaved structure of the enzyme-making specific to predict how a compound, such as *eupalitin 3-O-β-D-galactopyranoside* and captopril, interacts with that enzyme. Its docks with glycosides like galactopyranoside because the glycoside, intact, represents the native structure that may engage the target before hydrolysis in vivo. The sugar moiety may significantly have a role in the binding by differentially influencing affinity and specificity through hydrogen bonding or hydrophilic interactions. Docking with the glycoside, therefore, provides an insight into the possible biological relevance of that glycoside before interaction with body fluids, while hydrolysis would have occurred in those fluids. However, for a complete understanding, docking studies on the glycone could complement these findings. Based on the current calculations, molecular docking and binding affinity provide importance to these compounds to inhibit ACE as well as to develop new therapeutics for hypertension and cardiovascular diseases. The activity of these compounds as compared to captopril, which is a proven ACE inhibitor, elucidates the relative potency and potential viability of *eupalitin 3-O-β-D-galactopyranoside*. This finding suggests that eupalitin may function similarly to captopril by inhibiting ACE activity, thereby reducing the conversion of angiotensin I to angiotensin II and subsequently lowering blood pressure by vasodilation. This result is significant as it not only confirms the potential mechanism of action of eupalitin but also positions it as a competitive candidate in the realm of antihypertensive drugs. Captopril is a widely used ACE inhibitor with a well-documented therapeutic profile, and finding a natural compound with comparable activity underscores the value of exploring plant-derived substances for hypertension management. This research on molecular docking and antihypertensive activity evaluation of *eupalitin 3-O-β-D-galactopyranoside* from *Boerhavia diffusa* has certain limitations. First, it was confined to docking analysis of very few target proteins and ignored other mechanistic modules. Second, detailed toxicity profiles of the compound have not been investigated. Future studies should be experimental model-based and extensive profiling to corroborate these findings and assess clinical potential.

## 5. Conclusions

The study successfully demonstrated that *eupalitin 3-O-β-D-galactopyranoside*, isolated from *Boerhavia diffusa* Linn., exhibits significant antihypertensive activity. The compound’s efficacy in reducing blood pressure in hypertensive models highlights its potential as a natural therapeutic alternative for hypertension. UV spectroscopy proved to be a reliable method for quantifying eupalitin, ensuring accurate assessment of its concentration in the plasma. The molecular docking studies provided valuable insights into the interaction of *eupalitin 3-O-β-D-galactopyranoside* with the ACE protein. The comparable binding affinity of *eupalitin 3-O-β-D-galactopyranoside* to that of captopril suggests that *eupalitin 3-O-β-D-galactopyranoside* may act as a competitive inhibitor of ACE, like the well-established antihypertensive drug captopril. In summary, *eupalitin 3-O-β-D-galactopyranoside* shows promise as an antihypertensive agent with effective ACE inhibition. These findings lay the groundwork for future studies to explore its clinical potential, optimize its formulation, and further understand its mechanism of action. Future research should focus on detailed pharmacokinetics, long-term efficacy, and safety assessments to fully establish its role in hypertension management.

## Figures and Tables

**Table 1:** Animal experimental design.

Group	Treatment
I	Normal (normal saline 2 mL/kg/week)
II	Methyl predenisolone (20 mg/kg/week) as i.p.
III	Methyl predenisolone (20 mg/kg/week) as i.p + captopril (5 mg/kg, po.) on week
IV	Methyl predenisolone (20 mg/kg/week) as i.p + captopril (10 mg/kg, po.) on week
V	Methyl predenisolone (20 mg/kg/week) as i.p + *eupalitin 3-O-β-D-galactopyranoside* (10 mg/kg, po.) on week
VI	Methyl predenisolone (20 mg/kg/week) as i.p + *eupalitin 3-O-β-D-galactopyranoside* (10 mg/kg, po.) on week
VII	Methyl predenisolone (20 mg/kg/week) + *eupalitin 3-O-β-D-galactopyranoside* (40 mg/kg, po.) on week

Where i.p is the intraperitoneal administration, po. is oral administration.

**Table 2:** Docking scores (kcal/mol) for *eupalitin 3-O-Β-D-galactopyranoside* and standard captopril in the active site of PDB ID: 1J37. Captopril was used as a standard to judge the scoring potential of posing both peptide ligands in the active site of human angiotensin-converting enzyme (PDB ID: 1J37). Lower (more negative) docking scores indicate stronger binding affinities.

Sl. No.	Compound	Docking Score (kcal/mol)
1.	Captopril	−9.44
2.	*Eupalitin 3-O-* *β* *-D-galactopyranoside*	−7.89
3.	*Eupalitin 3-O-* *β* *-D-galactopyranoside*	−6.65
4.	*Eupalitin 3-O-* *β* *-D-galactopyranoside*	−6.58
5.	*Eupalitin 3-O-* *β* *-D-galactopyranoside*	−7.98

**Table 3:** The blood pressure variations in hypertensive-induced rats.

Group	Compound	Systolic BP	Diastolic BP	MABP (mmHg)	Heart Rate (Beats/min)
I	Normal (normal saline 2 mL/kg/week	122.33 ± 5.23	92.2 ± 6.11	102.94 ± 5.19	382 ± 8.56
II	Methyl predenisolone (20 mg/kg/week) i.p injection	198.44 ± 8.18	154.73 ± 7.19	167.96 ± 10.9 ##	641.66 ± 16.0
III	Methyl predenisolone 20 mg/kg/week) + oral captopril (5 mg/kg, single i.p injection on week	142.99 ± 10.88	117.8 ± 11.19	126.19 ± 11.10 *	507.11 ± 6.13
IV	Methyl predenisolone 20 mg/kg/week) + oral captopril (10 mg/kg, week	136.77 ± 6.13	98.66 ± 10.18	112.6 ± 5.17 **	421.78 ± 12.0
V	Methyl predenisolone 20 mg/kg/week) + *eupalitin 3-O-β-D-galactopyranoside* (10 mg/kg, single i.p injection on week	179.19 ± 7.18	126.99 ± 6.80	144.4 ± 4.59 *	443.99 ± 15.12
VI	Methyl predenisolone 20 mg/kg/week) + oral *eupalitin 3-O-β-D-galactopyranoside* (20 mg/kg on week	138.44 ± 7.09	110.88 ± 10.28	120.1 ± 5.1 **	430.66 ± 10.5
VII	Methyl predenisolone 20 mg/kg/week) + oral *eupalitin 3-O-β-D-galactopyranoside* (40 mg/kg, on week)	114.99 ± 5.8	85.76 ± 7.4	95.53 ± 7.5 **	408.99 ± 9.0

Where i.p is the intraperitoneal administration, n = 4, data presented as mean ± S.E.M. Comparisons were made between Groups III–VII and Group II using Dunnett’s post-hoc test. ** *p* < 0.01, * *p* < 0.05, ## denotes toxic significance.

**Table 4:** Pharmacokinetic parameters of eupalitin 3-O-β-D-galactopyranoside.

Eupalitin 3-O-β-D-galactopyranoside	AUC 0–7 (ng.h/mL)	Cmax (µg/mL)	MRT (h)	Elimination Rate (h^−1^)	AUM
10 mg/kg, b.w op	26,284.05	100 µg	182.9	0.000123585	4,80,9393
20 mg/kg, b.w. op	29,939.7	118.51 µg	178.5	0.000684634	5,34,4236
40 mg/kg, b.w, op	32,366.7	124.99 µg	182.3	0.000291677	5,90,0994

AUC: Area under the concentration-time curve; Cmax: Maximum plasma concentration; MRT: Mean residence time; AUM: Area under the moment curve.

## Data Availability

The data that supports the finding of this study are also available from the corresponding author upon request.
